# A 4-year Longitudinal Study of Well-being of Chinese University Students in Hong Kong

**DOI:** 10.1007/s11482-016-9493-4

**Published:** 2016-10-27

**Authors:** Daniel T. L. Shek, Lu Yu, Florence K. Y. Wu, Xiaoqin Zhu, Kevin H. Y. Chan

**Affiliations:** 1 Department of Applied Social Sciences, The Hong Kong Polytechnic University, Hunghom, Hong Kong, People’s Republic of China; 2 Centre for Innovative Programmes for Adolescents and Families, The Hong Kong Polytechnic University, Hong Kong, People’s Republic of China; 3 Department of Social Work, East China Normal University, Shanghai, People’s Republic of China; 4 Kiang Wu Nursing College of Macau, Macau, People’s Republic of China; 5 Division of Adolescent Medicine, Department of Pediatrics, Kentucky Children’s Hospital, University of Kentucky College of Medicine, Lexington, KY USA

**Keywords:** Life satisfaction, Engagement, Competence, Undergraduate study, Chinese students, Positive youth development

## Abstract

The present longitudinal study explored the development of personal well-being in university students over 4 years. Personal well-being was indexed by multiple indicators including life satisfaction, positive youth development qualities, and university engagement. A sample of 434 students enrolled in the new 4-year undergraduate program in one university in Hong Kong was successfully followed up for 4 years since they started their university study in 2012–2013 academic year. Students completed an online survey on a yearly basis and four waves of data were collected. Results revealed significant changes in most well-being indicators over time with three main observations. First, students’ life satisfaction remained at a stable level during 4 years. Second, most students’ self-reported positive youth development competencies followed a U-shaped developmental trajectory, which was characterised by a dip in the second year and a continuous rebound in the third year and fourth year. Third, students’ university engagement in different aspects showed significant increments in the third and fourth year of university study. The findings underscore the disparate developmental patterns of different aspects of student well-being during university study. This suggests that there is a need to take into account students’ developmental characteristics and related challenges in different stages of university life when develop and implement programs in university to promote student well-being.

## Introduction

Well-being, defined as the “optimal psychological functioning and experience” (Ryan and Deci 2001, p. 142), is important for one to actualize his/her full potential and achieve anticipated goals (Howell 2009). With the development of positive psychology in recent decades, well-being and related topics such as quality of life, happiness, and life satisfaction have gained considerable attention. In the field of youth development, well-being including positive affect and negative affect were closely related to adolescents’ personal functioning and outcomes (e.g., Fiedler and Beier 2014; Lewis et al. 2009). Similarly, life satisfaction as an overall cognitive evaluation of one’s quality of life (Ryan and Deci 2001) was negatively related to adolescent maladaptive outcomes such as hopelessness (Shek and Li 2016) and problem behaviors, including delinquency, substance abuse, and problematic Internet use (e.g., Birkeland et al. 2012; Sun and Shek 2012, 2013). Previous studies also highlighted positive associations of life satisfaction with school performance and academic achievement (e.g., Heffner and Antaramian 2015). While the importance of well-being in different aspects of youth’s life has been generally recognized and supported by empirical findings, how youth well-being develops over time remains controversial.

Previous studies found that adolescents tend to have moderate to high levels of life satisfaction (McCullough et al. 2000; Shek and Liu 2014), but there are age-related differences. For instance, based on participants from more than ten European countries, Michel et al. (2009) reported that adolescents had poorer quality of life than did children. Another study (Goldbeck et al. 2007) noted a significant declining trend in German adolescents’ life satisfaction in both general (e.g., family life, school life, and social relationships) and health-related domains (e.g., physical condition, freedom from anxiety, and ability to relax). Similar results showing that youth life satisfaction decreased over time were also reported in Asia, such as Hong Kong (Shek and Liu 2014) and South Korea (Park 2005). Some researchers thus considered decrease in life satisfaction during adolescence as a normal developmental phenomenon, which may in part result from notable changes and challenges that adolescents experience during the transition period from childhood to adulthood (Goldbeck et al. 2007; Shek and Liu 2014).

However, there were also studies showing that age was unrelated to youth life satisfaction and life satisfaction did not decline as adolescents matured (Suldo and Huebner 2004; Tian et al. 2014). More importantly, researchers noted that majority of the adolescents are capable of maintaining their adaptive functioning and personal well-being during adolescence (Birkeland et al. 2012; Warren et al. 2016). Besides inconsistent findings, there are mainly two methodological problems in the existing studies. First, as most studies used life satisfaction as the only indicator of well-being, we do not have any broader picture about changes in adolescent well-being in terms of other indicators, such as psychosocial competence and school engagement. Second, as most of the aforementioned studies are cross-sectional, whether youth well-being really changes over time is not definitive. While few studies have investigated the developmental trajectories of adaptive functioning based on secondary school students (e.g., Geldhof et al. 2014; Hilliard et al. 2014; Lewin-Bizan et al. 2010), different developmental trajectories (e.g., a continuous decrease trend, an increase followed by a decrease, or a stable increase trend) were reported which makes the findings inconclusive. It is worth to note that there are few studies examining the developmental patterns of well-being during late adolescence and early adulthood, a period ranging from late teens to early twenties. To fill these research gaps, the current study attempted to investigate longitudinal changes of well-being using multiple indicators in university students, who are regarded as experiencing late adolescence or early adulthood (Taylor et al. 2015).

### Conception of Well-being

Life satisfaction, together with the presence of positive emotions and the absence of negative emotions are three components of subjective well-being (SWB), which is considered as the primary index of well-being in existing literature (Ryan and Deci 2001). Also, the combination of the three components of SWB is often termed as happiness as a result of expectation and realization of desirable outcomes (Ryan and Deci 2001). Such measures tend to focus on broadened aspects of hedonic experience including not only physical hedonism but also pleasure of the mind and emotion (Ryan and Deci 2001). Although SWB measures have been predominantly used in well-being research field, some researchers have challenged the extent to which they adequately define wellness (Keyes et al. 2002). In particular, equating well-being with subjective pleasure and happiness could be invalid, because people can perceive themselves as well or in positive mood even when they are living an unhealthy life or suffering.

In this respect, some researchers argued that well-being consists of not only happiness but also fulfillment or actualization of one’s potential, goals and meaningful values (Ryan and Deci 2001). As stated by Ryff (1995), well-being is not only attaining desires, but also “the striving for perfection that represents the realization of one’s true potential” (p. 100). Such a view is often labeled as eudaimonism and the term “psychological well-being” (PWB) has been used as distinct from SWB (Ryan and Deci 2001). Researchers defined PWB in a multidimensional way to tap different facets of human actualization, such as self-development, personal growth, positive relations, and purposeful engagement (Ryff et al. 2004). According to this view, well-being is related to fully functioning rather than simply attaining pleasure and the use “happiness” are actually describing “psychological wealth” which is largely in agreement with PWB (Diener and Biswas-Diener 2011).

Based on the eudaimonic view, well-being can be measured from a broader framework under which any concepts related to one’s health functioning can be included as well-being indicators. For example, recent studies defined well-being as a health status in social, emotional, physical, and spiritual domains and measured it accordingly (Cloninger and Zohar 2011; Josefsson et al. 2011). Other research regarded one’s adaptability as an important marker of well-being, and used resilience (i.e., one’s competence in dealing with adversity and stress) as an indicator of well-being (Eley et al. 2013). With respect to youth health functioning, one influential model is the Five Cs Model, in which positive youth development (PYD) was defined in terms of competence, confidence, connection, character and caring (Bowers et al. 2010).

One “C” is competence which refers to “positive view of one’s actions in domain specific areas” (Bowers et al. 2010, p. 721), and this “C” has been comprehensively modeled by Catalano and colleagues (2002) after reviewing the critical components of effective PYD programs that could enhance young people’s all-round health status. Their competence model consisted of 15 PYD qualities, such as resilience, social competence, emotional competence, cognitive competence, self-determination, moral competence, and self-efficacy. There is extensive evidence suggesting that these PYD competencies are extremely important for adolescents to develop in a positive way and thus live a productive life and achieve prosperity (Catalano et al. 2012; Çelik et al. 2015; Sun and Shek 2013). Actually, some of the concepts incorporated in the PYD competence framework have been used in previous studies to index well-being, such as resilience used in Eley et al.’s (2013) research.

Notably, eudaimonic well-being is also derived from purposeful engagement and positive social relationships with others (Ryff et al. 2004). In agreement with this notion, the Five Cs Model also regards positive bonds with others and systems (e.g., school) as one important composition of youth health functioning. Given the important role of deep learning plays in attaining desired outcomes in university (Kilgo et al. 2015), purposeful engagement of students could be reflected by how they spend time and exert effort to engage in learning activities. Besides, in higher education context, relationships with peers, faculty members, and the institution as well, constitute important parts of university students’ social relationship (i.e., social competence). Taken these two points into consideration, one concept that may be able to cover the aforementioned dimension (i.e., “purposeful engagement and positive social relationships”) of well-being in university students is their school engagement, which represents how students adapt to higher levels of learning both independently and collaboratively, as well as how they interact with peers, teachers, faculty members and the institution (Kuh 2009c). In fact, previous studies have used school engagement and its related concept such as burnout to account for psychological well-being (e.g., Kotzé and Kleynhans 2013).

Although the two perspectives (i.e., the hedonic view and the eudaimonic view) of well-being may differ in how they define optimal experience and “what constitutes a good life”, studies utilizing the two different approaches tend to converge in their findings about how well-being is related to character traits and youth development outcomes. Specifically, indicators of SWB and PWB were both positively associated with a mature character profile (e.g., Cloninger and Zohar 2011) and favorable developmental outcomes (e.g., Kotzé and Kleynhans 2013). Besides, the two views are not independent. For example, eudaimonic personal fulfilment would certainly lead to subjective happiness, and empirical studies showed that while eudaimonic and hedonic well-being were distinct constructs, they mutually correlated with each other (see Keyes et al. 2002; McDowell 2010). In fact, recent studies tend to combine hedonic and eudaimonic aspects to form a holistic measure of well-being. For example, Cloninger and Zohar (2011) measured well-being from affective aspects (e.g., happiness) and non-affective aspects (e.g., social support, life satisfaction, and physical health). Thus, to demonstrate a comprehensive picture of developmental pattern of well-being, it is necessary to embrace a broad conception of well-being, including measures of SWB and PWB. As a result, the present study used life satisfaction, PYD qualities such as psychosocial competencies, and school engagement to indicate university students’ well-being.

### Development of Well-being in University Students

As mentioned, another research gap in well-being research field is that few studies adopted longitudinal research design to trace the development of well-being in youth, especially in late adolescence and young adulthood. While university can be regarded as a transition period from late adolescence to early adulthood, less attention has been paid to how well-being of university students changes during their university lives. Similar to middle adolescence, late adolescence and early adulthood are also characterized by considerable difficulties and challenges (e.g., Gress-Smith et al. 2015; Shek and Wong 2011). Autonomous and less structured learning and living environment of university life requires students to function more independently than does the securer and more supportive high school environment. More specifically, university students need to adjust to new life style, fulfill demanding academic requirements, spend more time and energy in deep and independent learning and deal with new social relationships, all of which may lead to experiences that are physically, emotionally and psychologically stressful (Wynaden et al. 2013). It was argued that such academic, environmental and social pressures may harm students’ academic success and make them at risk of developing psychosocial problems (Tobolowsky 2008; Wynaden et al. 2013). In fact, increasing psychological and behavioral problems of students in their first few years of university study were reported in both Western countries and Chinese cultures (e.g., Song et al. 2008; Walther et al. 2012). Relating these findings to well-being, it is reasonable to expect that university students might experience a decline in their well-being during the difficult transition period in university. However, it is a common myth that adolescent problems would disappear after they enter universities (Shek and Wong 2011). As a result, the current study attempted to trace well-being of university students in the Chinese context, where very few related studies have been conducted.

Studies in positive psychology suggest that keeping a high level of well-being is vital for students to achieve success in academic domains and beyond (Marks and Wade 2015). Recognizing the importance of promoting student well-being, institutions related to higher education have invested massive resources in developing and implementing credit-bearing subjects and non-credit bearing programs to enhance university students’ well-being. For example, most higher education institutions in the US have incorporated “higher-impact educational practices” (e.g., freshmen seminar, learning communities) in their general education (GE) curriculum. “Higher-impact educational practices” represent different forms of teaching and learning practices that aim to help students reap the most benefits from higher education (Kuh 2009a). These practices include “first-year seminars and experiences”, “learning communities”, “collaborative assignments and projects”, “service learning and community based learning”, and so on (Kuh 2009a). In the US, “higher-impact educational practices” incorporated in GE have been widely examined and regarded as having positive contributions to students’ personal growth and well-being (e.g., Kilgo et al. 2015; Pike et al. 2011). These findings supported the effectiveness of “high-impact practices” in promoting students’ well-being as well as demonstrating that students’ well-being may be improved due to university learning and subsequent personal gains. However, similar higher education practices are grossly lacking in Chinese contexts.

With specific reference to Hong Kong, the higher education system has experienced rapid development and significant reform during past few years. In the academic year of 2012/2013, the University Grants Committee (UGC) in Hong Kong mandated the extension of 1 year for undergraduate education. As a result, previous British framework of 3-year undergraduate education was transformed to a 4-year structure, of which the extra 1 year (usually the first year) could be devoted to implementing GE curriculum. A particular example is The Hong Kong Polytechnic University (PolyU), one university that has refined its undergraduate education and implemented a newly developed GE curriculum entitled “General University Requirements” (GUR) since 2012–2013 academic year (Shek et al. 2014b). Aiming to facilitate student holistic development, the GUR includes diverse components, such as “Freshmen Seminar”, “Service Learning”, “Language and Communication Requirements”, and “Leadership and Intrapersonal Development” (LIPD), in which the experiential teaching and learning pedagogy is commonly utilized (Shek et al. 2014b). Furthermore, some credit-bearing leadership courses are specifically designed based on the PYD approach to promote competencies in students (Shek 2012; Shek et al. 2013). All these characteristics of GUR coincide with the aforementioned “higher-impact educational practices” in the US.

It is expected that the new 4-year undergraduate curriculum in Hong Kong would nurture qualified graduates with holistic competencies and promote students’ well-being as well (Freake 2013). Several evaluation studies have showed that GE study had positive effects on students’ well-being in terms of development of PYD attributes and increased life satisfaction (Shek and Sun 2012; Shek et al. 2014a). However, whether there will be positive changes in students’ well-being as a result of university study (including GE study) is not clear. In other words, there is a great need to investigate students’ well-being throughout the university life and explore whether there are positive changes in different aspects of well-being as a result of university study experience.

Against this background, the present study attempted to investigate well-being of Hong Kong students during the period of their university study with regard to several aspects of well-being, including life satisfaction, PYD qualities, and school engagement. Particularly, the current study explored changes of students’ well-being in one university in Hong Kong (i.e., PolyU). A simple pretest-posttest design with three posttests was adopted. Student well-being was examined when they enrolled in the university (i.e., pretest), and followed up each year afterwards (i.e., posttests).

To sum up, the current study addressed two research questions. The first question is that how student well-being changes during university life. Despite of the huge challenges in late adolescence and early adulthood (Shek and Wong 2011), mounting evidence has suggested that students could benefit a lot from good practices in general education during university life (Kuh 2009a; Shek et al. 2014a, b, 2015). As a result, we expected growth in student well-being. The second question to be explored in current study is that whether there are any differences in the growth pattern with respect to different indicators of well-being. Due to lack of related evidence in previous research, we did not make any specific hypotheses regarding this research question.

## Methods

### Participants and Procedures

In order to examine changes of students’ well-being over time, a randomly selected cohort of students enrolled in the new 4-year undergraduate program in one university in Hong Kong was followed up since they started their university study in the academic year of 2012–2013. Before the first wave (i.e., Wave 1) of data collection, 650 freshmen were randomly selected and invited to participate in a longitudinal study in which they need to complete an online survey on a yearly basis during university study. The students were informed that the aim of the study was to investigate how a new GE program will influence students’ well-being and all information they provided would be kept confidential and only used for purpose of scientific research. Informed consent was obtained from students after they agreed to participate in the study.

The first wave of data collection was conducted in November 2012. A total of 543 out of 650 invited students completed the survey, resulting in a response rate of 83.54 %. The data included participants’ demographic information (e.g., gender, age, and place of birth) and initial status of well-being reflected by several measures including life satisfaction, PYD qualities, and university engagement. After Wave 1 data collection, those 543 participants were followed up each year in November and another three waves of data have been collected up until 2015 (i.e., Wave 2: November 2013; Wave 3: November 2014; Wave 4: November 2015). In each wave of follow-up data collection, participants completed the same online survey containing measures of well-being.

In total, we successfully matched 434 participants (*M*_*age*_ = 18.13, *SD*_*age*_ = .54 at Wave 1) across four waves, suggesting a low average attrition rate of 5.02 % across 4 years. This attrition rate compared favorably with other longitudinal studies (e.g., Dion et al. 2016; King et al. 2015; Scales et al. 2006). There were no significant differences between the matched sample (*n* = 434) and those students who dropped out of the study after Wave 1 (*n* = 109) in age, gender composition, place of birth, and initial score in measures of well-being. Among the matched 434 participants, 266 (61.29 %) were female and 168 (38.71 %) were male. All these participants were Asian, and 314 (72.35 %) of them were born in Hong Kong, 106 (24.42 %) were born in mainland China, and the remaining 14 (3.23 %) participants were born in other places in Asia.

### Well-being Measures

To tap different aspects of well-being, we used three core instruments that include several measures. The core instruments were Satisfaction with Life Scale (SWLS), Chinese Positive Youth Development Scale (CPYDS) and National Survey of Student Engagement (NESS).

#### Satisfaction with Life Scale (SWLS)

The present study adopted the Satisfaction with Life Scale (SWLS) to assess students’ global assessment of their quality of life. There were five items in the scale measured on a 6-point Likert scale (1 = strongly disagree, 6 = strongly agree), with a higher score indicating higher life satisfaction. The scale showed good reliability in previous studies investigating Chinese adolescents’ life satisfaction (Shek and Li 2016; Shek and Liu 2014). In the present study, the Cronbach’s alpha coefficients ranged from .77 to .81 across four waves (see Table 1), indicating good internal consistency of the scale.

**Table 1:** Reliability analyses of well-being measures

Instruments	Indicators	# of items	Cronbach’s α				Mean inter-item correlations			
			Wave 1	Wave 2	Wave 3	Wave 4	Wave 1	Wave 2	Wave 3	Wave 4
SWLS	Life satisfaction	5	.86	.86	.89	.91	.58	.57	.63	.69
CPYDS	Resilience	3	.77	.78	.79	.81	.52	.54	.54	.58
	Social competence	3	.85	.86	.88	.86	.66	.67	.71	.69
	Emotional competence	3	.68	.74	.72	.73	.42	.50	.47	.47
	Cognitive competence	4	.70	.75	.77	.78	.39	.45	.46	.49
	Behavioral competence	2	.44	.60	.62	.63	.28	.43	.45	.47
	Moral competence	3	.64	.66	.69	.70	.38	.39	.42	.44
	Self-determination	2	.67	.75	.80	.74	.51	.60	.68	.60
	Self-efficacy	2	.49	.60	.50	.61	.32	.40	.34	.44
NSSE	Reflective and integrative learning	7	.82	.84	.86	.86	.40	.43	.48	.48
	Higher order learning	4	.77	.77	.80	.81	.45	.46	.49	.51
	Quantitative reasoning	3	.70	.70	.76	.82	.44	.44	.52	.60
	Learning strategies	3	.70	.74	.77	.77	.43	.49	.53	.53
	Collaborative learning	4	.70	.75	.76	.79	.36	.42	.44	.48
	Discuss with diverse other	4	.67	.63	.69	.72	.34	.30	.36	.39
	Student-faculty interaction	4	.76	.80	.84	.87	.44	.51	.57	.62
	Effective teaching practice	5	.75	.74	.81	.83	.37	.36	.46	.49
	Quality of interaction	5	.83	.87	.85	.88	.50	.57	.52	.60
	Supportive environment	8	.87	.87	.88	.90	.44	.46	.49	.52

*SWLS* Satisfaction with Life Scale; *CPYDS* Chinese Positive Youth Development Scale; *NSSE* National Survey of Student Engagement

#### Chinese Positive Youth Development Scale (CPYDS)

Developed by Shek et al. (2007), the 90-item Chinese Positive Youth Development Scale (CPYDS) comprised 15 subscales corresponding to the 15 constructs of positive youth development attributes proposed by Catalano and his colleagues (2002). The CPYDS has been widely used to measure Chinese adolescents’ PYD attributes and demonstrated sound validity and reliability in previous studies (e.g., Shek and Ma 2010; Sun and Shek 2013). The present study used 8 out of the 15 subscales to measure university students’ PYD competencies: Resilience (RE), Social Competence (SC), Emotional Competence (EC), Cognitive Competence (CC), Behavioral Competence (BS), Moral Competence (MC), Self-Determination (SD), and Self-Efficacy (SE). A 6-point Likert scale was used for all CPYDS items with a higher score representing a higher level of competence. Average scores across items in each subscale were calculated and used for analyses. In the present study, all subscales had acceptable to good reliability across four waves as shown in Table 1.

#### National Survey of Student Engagement (NSSE)

One well-acknowledged operational model of measuring student engagement at college level is developed through conducting nation-wide annual survey in the US (i.e., NSSE) (Kahu 2013; Kuh 2009b). This model applied several Engagement Indicators (EIs) to comprehensively measure university engagement (National Survey of Student Engagement 2015), and has been transplanted to other countries, including China. The NSSE-China, which was culturally adapted and renamed as “Chinese College Student Survey” (CCSS) has been conducted annually since 2010 (Ross et al. 2014; Wang 2011). After revision in 2012, the NSSE consisting 47 items that were mapped into 10 EIs, which assessed student engagement in high levels of learning as well as their interactions with peers, teachers, and faculty members. The 10 EIs included Reflective and Integrative Leanring (RIL), Higher Order Learning (HO), Quantitative Reasoning (QR), Learning Strategies (LS), Collaborative Learning (CL), Discuss with Diverse Other (DD), Student-faculty Interaction (SFI), Effective Teaching Practice (ETP), Quality of Interaction (QI), and Supportive Environment (SE). In the current study, except for items in QI subscale which were measured on a 7-point Likert scale, items of other 9 EIs all used a 4-point Likert scale. An average score of each EI was computed as an indicator of students’ engagement and higher scores indicated higher levels of engagement. All the 10 EIs showed good reliabity in the present study (see Table 1).

## Results

To explore students’ well-being development across years, we examined how each indicator of well-being changed across four waves. For life satisfaction, a repeated measures analysis of variance (ANOVA) was performed, using time operationalized as four waves as the independent variable. For PYD competence and university engagement, one repeated measures multivariate analysis of variance (MANOVA) followed by univariate tests was conducted for each aspect using corresponding indicators as dependent variables and also time (i.e., four waves) as the independent variable.

### Life Satisfaction

As shown in Table 2, students were overall satisfied with their life when they started their university life (mean = 3.83 at Wave 1 in a 6-point scale), and their life satisfaction did not show significant variation in the following 3 years (*F* (3, 1299) = 1.93, *p* = .13).

**Table 2:** Comparisons of students’ well-being across four waves (*n* = 434)

Indicators	Wave 1		Wave 2		Wave 3		Wave 4		Repeated Measures MANOVA		
	*M*	*SD*	*M*	*SD*	*M*	*SD*	*M*	*SD*	F	*η* ^*2*^_*p*_	Pairwise comparisons
Life satisfaction	3.83	.93	3.85	.90	3.88	.93	3.92	.99	1.93	.004	
Positive youth development competence									2.72^***^	.02	
Resilience	4.72	.75	4.57	.77	4.62	.77	4.59	.78	6.51^***^	.02	W1 > W4 &W3 & W2
Social competence	4.58	.77	4.58	.73	4.61	.75	4.64	.73	1.07	.002	
Emotional competence	4.40	.71	4.28	.71	4.34	.72	4.40	.71	5.23^**^	.01	W4 & W1 > W2
Cognitive competence	4.60	.58	4.50	.62	4.56	.60	4.59	.63	4.32^**^	.01	W4 & W1 > W2
Behavioral competence	4.50	.67	4.45	.71	4.55	.70	4.57	.73	5.15^**^	.01	W4 & W3 > W2
Moral competence	4.80	.65	4.72	.67	4.79	.67	4.82	.67	3.62^*^	.01	W4 & W3 > W2; W1 > W2
Self-determination	4.50	.75	4.44	.74	4.52	.76	4.57	.72	4.41^**^	.01	W4 >W2
Self-efficacy	4.51	.78	4.43	.80	4.51	.75	4.54	.74	2.47^†^	.01	W4 > W2
University engagement									17.07^***^	.12	
Reflective and integrative learning	2.34	.48	2.28	.47	2.64	.51	2.63	.50	110.66^***^	.20	W4 & W3 > W2 & W1
Higher order learning	2.43	.57	2.50	.56	2.77	.56	2.77	.56	60.05^***^	.12	W4 & W3 > W2 & W1
Quantitative reasoning	2.06	.56	2.15	.56	2.40	.60	2.48	.69	67.07^***^	.13	W4 & W3 > W2 > W1
Learning strategies	2.50	.62	2.46	.57	2.60	.60	2.55	.59	7.13^***^	.02	W4 & W3 > W2, W3 > W1
Collaborative learning	2.43	.55	2.39	.53	2.60	.54	2.55	.55	22.19^***^	.05	W4 & W3 > W2 & W1
Discuss with diverse other	2.28	.55	2.24	.52	2.46	.58	2.37	.57	23.68^***^	.05	W3 > W4 > W2 & W1
Student-faculty interaction	1.65	.51	1.71	.58	1.96	.66	1.99	.71	57.11^***^	.12	W4 & W3 > W2 & W1
Effective teaching practice	2.53	.52	2.51	.49	2.71	.52	2.70	.52	25.96^***^	.06	W4 & W3 > W2 & W1
Quality of interaction	4.55	.85	4.63	.90	4.64	.82	4.69	.85	3.40^*^	.01	W4 > W1
Supportive environment	2.42	.55	2.37	.52	2.52	.56	2.50	.57	11.22^***^	.03	W4 & W3 > W2 & W1

^†^ *p* < .10; ^*^ *p* < .05; ^**^ *p* < .01; ^***^ *p* < .001

### Positive Youth Development (PYD) Competence

Repeated measures MANOVA revealed a significant difference of participants’ PYD competence across four waves (Wilks’ λ = .95, *F* (24, 3747) = 2.72, *p* < .001, *η* ^*2*^_*p*_ = .02). Results of further univariate tests are presented in Table 2. As shown in Table 2, among the eight indicators of PYD attributes, apart from SC that did not have significant changes across four waves (*F* (3, 1299) = 1.07, *p* = .36) and SE that showed marginally significant changes (*F* (3, 1299) = 2.47, *p* = .06, *η* ^*2*^_*p*_ *=* .01), other six measures (i.e., RE, EC, CC, BC, MC, SD) all demonstrated significant changes over time with partial effective size ranging between .01 and .02. Post-hoc pairwise comparisons further revealed that students had highest level of resilience at Wave 1 (*M* = 4.72, *SD* = .75), and this score was higher than that at Wave 2 to 4 (*F* (3, 1299) = 6.51, *p* < .001, *η* ^*2*^_*p*_ *=* .02). For EC, CC, and MC, there was a significant decrement in students’ scores from Wave 1 to Wave 2, but students’ performance in these three dimensions rebounded continuously from Wave 2 to Wave 4, with the scores at Wave 4 significantly higher than that at Wave 2. For BC, SD, and SE, although students’ scores also seemed to have a decrease trend from Wave 1 to Wave 2, the decrement did not reach a significant level. Similarly, students’ scores in these three measures also showed a steady and continued increasing after Wave 2, with self-perceived performance at Wave 4 significantly better than that at Wave 2.

### University Engagement

Repeated measures MANOVA also revealed a significant difference for students’ engagement across four waves (Wilks’ λ = .69, *F* (30, 3787) = 17.07, *p* < .001, *η* ^*2*^_*p*_ = .12). Results of following univariate tests are also demonstrated in Table 2. These results indicated a significant main effect of time on all measures of students’ engagement, with partial effective size ranging from .01 to .20. Post hoc comparisons revealed that student engagement remained stable during the first year of study, as student EI scores at Wave 1 and Wave 2 did not show significant differences. Besides, students tended to show better engagement at Wave 3. For instance, on all but one EI (i.e., QI), students’ scores were significantly higher at Wave 3 compared with their scores at Wave 1 and 2. However, there was an increasing tendency in students’ score on QI from Wave 1 to Wave 3, although the tendency did not reach significant level. Nevertheless, students showed significantly higher QI score at Wave 4 compared with their situation at Wave 1. Furthermore, students had relative high level of engagement at Wave 4, which was not significantly different from that at Wave 3, except for the engagement indicator of DD. Specifically, students discussed with diverse others less often at Wave 4 than at Wave 3. Concerning changes from Wave 1 to Wave 4, students did not show significant improvement on only one indicator, i.e., Learning Strategies. In other words, students’ scores on other nine EIs (i.e., RIL, HO, QR, CL, DD, SFI, ETP, QI, and SE) increased significantly from Wave 1 to Wave 4.

## Discussion

Using four-wave longitudinal data collected in one university in Hong Kong, the present study investigated the development of university students’ well-being in terms of life satisfaction, positive youth development (PYD) competencies, and university engagement. There are several unique features of this study. First, a longitudinal design was used which can capture changes over time. Second, this is the first known scientific study that tracks the development of Chinese university students. Third, a wide range of indicators were used to understand changes in well-being in Chinese university students.

As an important and perhaps the most investigated component of well-being, life satisfaction of adolescents has been found to decline with age in some previous studies (e.g., Shek and Liu 2014) but was moderately stable in other studies (e.g., Suldo and Huebner 2004). While these studies had a specific focus on middle school students who are in a critical transition period from childhood to adulthood, the present study examined life satisfaction of university students, who are also entering a new stage of life when they should prepare themselves for future career.

The present finding of students’ stable life satisfaction over university years implies that university students are able to maintain a satisfactory feeling of their life, even though they need to cope with different challenges. Although undesirable things and stressful life are likely to impair students’ life satisfaction, support from school, faculty and family as well as their enhanced ability as a result of university learning could help them deal with life stress, and buffer negative impacts of stressful environment on life satisfaction (Kong et al. 2012). This reasoning is in line with some researchers’ suggestion that adolescents will have reasonable understanding of their current situation and future development as they grow older and become mature (Shek and Li 2016).

With respect to the U-shaped changing pattern of students’ PYD competencies, it is characterized by a decline in second year and a steady rebound in the following years. The decline trend of competence is consistent with our expectation that students may have lower level of well-being due to typically stressful first-year transition which is characterised by changing educational, social and emotional demands of higher education. In dealing with these challenges, first-year students, who have not yet possessed sufficient independence and optimal coping strategies in learning and living, are likely to experience unfavourable comparisons, adverse outcomes and negative emotions (Wynaden et al. 2013). These experiences may cause identity confusion and underestimated self-evaluations (Scanlon et al. 2007; Tobolowsky 2008), which may subsequently result in lower level of self-evaluated competencies. Nevertheless, student life satisfaction remained at a moderate level instead of declining along with the decrease of self-evaluations. Noting that students only had significant lower evaluations on some aspects of competence, such a decline might not be great enough to threaten students’ overall life satisfaction, which represents a global evaluation of life quality.

Although pressure typically being recognized during first-year could continue into the following years and there may be new difficulties and challenging circumstances (e.g., advanced demands in major learning, more interactions with the environment outside school) after first-year transition (Stamp et al. 2015), students are likely to have learned effective coping strategies and even thrive amidst the challenges they encountered in later years of university life. This is possibly the reason that students’ ratings of their own competence rebounded significantly in the third and fourth year. This speculation is indirectly supported by previous findings suggesting that students could perform better and more effective learning in later years than in first year of university life (e.g., Roohr et al. 2016). Besides, such a developmental trend of university study is further supported by the present finding of a steady growth trend in students’ university engagement after first year. This U-shaped relationship of PYD qualities over time is novel in the scientific literature, and this pioneering finding is certainly insightful for future studies to investigate developmental trajectory of adolescents’ well-being over even a longer period.

In scientific literature, there is a heated discussion about student engagement with regard to its antecedents and associations with personal developmental outcomes (Upadyaya and Salmela-Aro 2013). On top of previous studies, the present study highlights the longitudinal change pattern of engagement in university students. Basically, after 2 years of university study, students not only performed better in independent and deep learning as well as collaborative learning with peers, but also interacted more effectively with faculty members, teachers and other staff in the campus (e.g., academic advisors and student service staff). Certainly, such an improvement in learning and utilizing social support would positively contribute to students’ adaptive coping and development of generic competence (Upadyaya and Salmela-Aro 2013) as well as life satisfaction (Lewis et al. 2011; Yuen 2016). Therefore, increased engagement after first few years of transition to higher education may partially, if not fully, explain the rebound of students’ evaluations of their competence after Wave 2. However, enhanced psychological well-being in terms of active engagement and improved competence did not extend to life satisfaction. As subjective life satisfaction is a broader concept that reflects complex interactions between individual factors and environmental factors (Diener et al. 2012), increasing in engagement and competence may not be great enough to promote global life satisfaction, just as the decline in competence during second year did not occur along with a decline in life satisfaction. Nevertheless, the present findings suggest that future studies need to compare the developmental trends of life satisfaction and of other well-being aspects.

Our findings of variations in PYD competencies and school engagement as indicators of psychological well-being supported the notion that “eudaimonic well-being is…dynamic, showing cross-time change as individuals negotiate particular life transitions” (Ryff et al. 2004, p. 1384). These findings shed light on how different aspects of well-being (e.g., subjective well-being vs. psychological well-being) develop differently over time in university students, and will add important values to existing scientific literature on well-being. Despite the different patterns over 4 years, one shared feature is that students’ well-being tended to improve in their fourth year of university life as compared with the baseline condition at Wave 1, although the increment did not reach to a significant level in some indicators (e.g., life satisfaction). To some extent, this observation supports our expectation that there will be a growth in student well-being due to good teaching and learning practices incorporated in general education. In other words, the positive changes in student well-being observed in the current study could be partially due to GUR impact. This interpretation is in line with previous evaluation findings (e.g., Shek and Sun 2012; Shek et al. 2014a, b, 2016), which suggest that students enjoy GUR study very much and demonstrate favorable changes after taking GUR subjects.

Besides theoretical values, students’ developmental trends of life satisfaction, PYD competencies, and engagement found in the present study also provide practical insights for designing and implementing student counseling service and PYD programs in university. In first-year transition, students face many adjustment difficulties that may lead to a decrease in perceived PYD competencies. Thus, to help students better adapt to university life and develop desired attributes, it would be necessary to initiate programs or courses targeting first-year students. In particular, helping students realize their strength in different domains of school life as well as guiding them to make proper psychological adjustment would help students maintain reasonable self-evaluations. In fact, previous evaluation studies have found that leadership courses incorporating PYD theories could enhance students’ competence and life satisfaction (Shek and Sun 2012; Shek et al. 2014a). Besides, students will be better engaged in collaborative and deep learning in later years of university life and their self-evaluations would rebound as well. However, such an increase found in present study is not great enough to bring about a similar increase in students’ life satisfaction. Therefore, programs with an objective to enhance student engagement could be carried out in early years of university life, and later years as well. After all, numerous studies have shown that active and positive engagement were associated with multiple desired life outcomes and life satisfaction (Upadyaya and Salmela-Aro 2013; Yuen 2016).

The present study represents a pioneering effort to explore the development of university students’ well-being through a 4-year longitudinal study in Chinese culture. However, the study has several limitations. First, data was only collected in one university in Hong Kong due to difficulties in following up students over multiple years. Future studies could involve a larger sample of students coming from different higher education institutions in Hong Kong and abroad, so that more conclusive findings could be generated and generalized to a broader population. Second, we only examined global life satisfaction. Future studies could further investigate students’ satisfaction with specific life domains such as school life and social relationships to explore any potential differences in developmental trends between global life satisfaction and domain specific life satisfaction. Third, although we have measured well-being in a comprehensive way, there are still other indicators of well-being not being included, such as purpose in life and personal growth (Keyes et al. 2002; Ryff et al. 2004). Fourth, the present study only described the developmental pattern of university students’ well-being, and more studies shall be conducted to examine factors that may affect the developmental trajectory.

Despite these limitations, this pioneering study enriches the literature on the developmental trend of youth well-being, particular with respect to university students under a new 4-year undergraduate curriculum within a Chinese context. Different changing patterns of different aspects of well-being highlight the importance of measuring well-being with comprehensive indicators. Furthermore, the accumulation of knowledge about students’ self-evaluation of their competence, satisfaction with life, and university engagement can help youth workers and educators to develop and implement tailored prevention and intervention programs to promote university students’ well-being.
